# Evaluation of breath, plasma, and urinary markers of lactose malabsorption to diagnose lactase non-persistence following lactose or milk ingestion

**DOI:** 10.1186/s12876-020-01352-6

**Published:** 2020-06-29

**Authors:** Aahana Shrestha, Matthew P. G. Barnett, Jo K. Perry, David Cameron-Smith, Amber M. Milan

**Affiliations:** 1 The Liggins Institute, The University of Auckland, Auckland, New Zealand; 2 The Riddet Institute, Palmerston North, New Zealand; 3 Food Nutrition & Health Team, AgResearch Limited, Palmerston North, New Zealand; 4 The High-Value Nutrition National Science Challenge, Auckland, New Zealand; 5 Singapore Institute for Clinical Sciences, Agency for Science, Technology and Research, Singapore, Singapore

**Keywords:** Lactose malabsorption, Single nucleotide polymorphism, Urinary galactose, Breath H_2_, Milk

## Abstract

**Background:**

Adult lactase non-persistence (LNP) is due to low lactase expression, resulting in lactose malabsorption (LM). LNP is a genetic trait, but is typically determined by LM markers including breath H_2_, blood glucose, and urinary galactose after a lactose tolerance test. Known validity of these markers using milk is limited, despite being common practice. Compositional variation, such as β-casein variants, in milk may impact diagnostic efficacy. This study aimed to evaluate the diagnostic accuracy to detect LNP using these commonly measured LM markers after both lactose and milk challenges.

**Methods:**

Fourty healthy young women were challenged with 50 g lactose then randomized for separate cross-over visits to ingest 750 mL milk (37.5 g lactose) as conventional (both A1 and A2 β-casein) and A1 β-casein-free (a2 Milk™) milk. Blood, breath and urine were collected prior to and up to 3 h following each challenge. The presence of C/T_13910_ and G/A_22018_ polymorphisms, determined by restriction fragment length polymorphism, was used as the diagnostic reference for LNP.

**Results:**

Genetic testing identified 14 out of 40 subjects as having LNP (C/C_13910_ and G/G_22018_). All three LM markers (breath H_2_, plasma glucose and urinary galactose/creatinine) discriminated between lactase persistence (LP) and LNP following lactose challenge with an area under the receiver operating characteristic (ROC) curve (AUC) of 1.00, 0.75 and 0.73, respectively. Plasma glucose and urinary galactose/creatinine were unreliable (AUC < 0.70) after milk ingestion. The specificity of breath H_2_ remained high (100%) when milk was used, but sensitivity was reduced with conventional (92.9%) and a2 Milk™ (78.6%) compared to lactose (sensitivities adjusted for lactose content). The breath H_2_ optimal cut-off value was lower with a2 Milk™ (13 ppm) than conventional milk (21 ppm). Using existing literature cut-off values the sensitivity and specificity of breath H_2_ was greater than plasma glucose to detect LNP following lactose challenge whereas values obtained for urinary galactose/creatinine were lower than the existing literature cut-offs.

**Conclusion:**

This study showed accurate diagnosis of LNP by breath H_2_ irrespective of the substrate used, although the diagnostic threshold may vary depending on the lactose substrate or the composition of the milk.

**Trial registration:**

ACTRN12616001694404. Registered prospectively on December 9, 2016.

## Background

Lactose, the predominant disaccharide in milk, is readily hydrolysed by the small intestinal enzyme lactase, liberating the constituent glucose and galactose [1]. However, in the majority of the non-northern European population, the expression of this enzyme is suppressed in early childhood, resulting in lactase non-persistence (LNP) [2]. With lactose ingestion, LNP results in lactose malabsorption (LM), and in some individuals, this undigested lactose contributes to the adverse digestive symptoms causing lactose intolerance (LI) [3].

LI can be avoided through exclusion of dietary lactose; however, intolerance to other foods, such as short chain carbohydrates and polyols (fermentable oligosaccharides, disaccharides, monosaccharides and polyols, or FODMAPs) [4] or proteins like gluten [5] or casein [6], can also result in similar digestive symptoms such as bloating, flatulence, diarrhoea, abdominal pain, rumbling, and distension. Therefore, confirmation of LM is required to limit unnecessary avoidance of dairy foods, given their importance for a balanced diet [1, 7] and the potential for micronutrient deficiency with dairy avoidance [8]. Different methods are currently used to diagnose LNP, of which measuring lactase activity in an intestinal biopsy is the proposed “gold standard” [9, 10]. However, this is an invasive technique for a relatively minor condition. Other more readily measurable, cost-effective and less invasive methods are therefore preferred [11, 12].

The minority of the adult population maintains lactase expression, known as lactase persistence (LP). Defined genetic polymorphisms have been identified which are associated with continued lactase expression. For example, two single nucleotide polymorphisms (SNPs) upstream from the *LCT* locus (C/T_13910_ and G/A_22018_) of the minichromosomal maintenance complex component 6 (MCM6) gene are highly associated with LP [13]. Homozygotes (TT_13910_ and AA_22018_) or heterozygotes (CT_13910_ and GA_22018_) show continued lactase activity (LP) whereas the wild type (CC_13910_ and GG_22018_) results in a loss of lactase activity (LNP), as demonstrated in primarily Caucasian populations [14, 15]. Genotyping these SNPs enables accurate diagnosis of LNP and is preferable to intestinal biopsy; however, genotyping remains expensive and has limited applicability to populations and ethnicities for whom LNP-associated polymorphisms may vary [16]. Genotyping is also ineffective for secondary LM, sometimes present with gastrectomy [17] or other gastrointestinal diseases including celiac disease [18] and Crohn’s disease [19] where the cause is damage to the intestine rather than genetic alteration of lactase expression [20].

Other indirect methods used include measurement of breath hydrogen (H_2_; which indicates fermentation of undigested lactose from colonic bacteria [21, 22]), blood glucose and urinary galactose, indicating hydrolysis of lactose. An incremental rise in breath H_2_ above 20 ppm [23–25] and reduction of plasma glucose below 1.11 mmol/L following lactose challenge [12, 26] indicate LM or LNP. Urinary galactose is frequently reported using a variety of testing procedures and reporting methods [27–30] limiting direct comparisons within the literature. Grant et al. [28] proposed that a urinary galactose/creatinine ratio after pooled 3 h collection provided the best discrimination; on this basis, a threshold of 0.1 mg/mg has been applied by subsequent studies as an absolute threshold [27, 28, 30] or lactose dose extrapolation [31]. Although these indirect measures of LNP depend on the lactose dose [23, 32] and substrate used (e.g. milk) [32], validity and cut-off value specific to the substrate and dose have not been validated [32, 33].

Lactose in its pure form or present in milk may be digested differently [34] but is used interchangeably to diagnose LM [25, 32, 33, 35, 36]. For consumers, milk is more accessible than purified lactose, and with the availability of personal devices like AIRE to detect LM [37], may be a simple method for at-home testing. Moreover, it has been argued that milk is a more physiologically relevant and realistic substrate than lactose for assessing LM [32, 36]. Milk has been used as a substrate in a variety of studies to establish LM [25, 32, 33, 35, 36]. However, LM and LI symptoms may be influenced by the food matrix or rate of digestion [38] thus, digestive responses may vary between isolated lactose and milk. The presence of other components in milk such as fats [32] may reduce gastric emptying thus influencing LM markers [32, 34]. More importantly, diagnostic thresholds of LM measures using milk have not been validated [32, 33]. Recent studies have also highlighted that milk protein variants arising from species [39] or breed differences [40] may impact digestion and malabsorption. Notably, the predominant β-casein variants, A1 and A2 β-casein, are hypothesised to differentially impact LM [31, 35], suggesting that LM markers may perform differently depending on milk protein, or specifically A1 β-casein, content.

This study aimed to establish the diagnostic accuracy of LM measures (breath H_2_, plasma glucose, and urinary galactose/creatinine) to diagnose LNP using genotyping (LCT C/T_13901_ and G/A_22018_ SNPs) as a reference method following both lactose and milk challenges. Furthermore, the study aimed to establish diagnostic thresholds following milk with differing β-casein types.

## Methods

### Study design

We conducted a double blinded randomized cross over study in healthy young women aged 18–30 years. The primary outcome of the study, reported elsewhere, was to investigate digestive comfort experienced by dairy intolerant individuals following ingestion of lactose and milk with differing bovine β-casein variants [41]. In total, 40 young women were recruited from the community, 30 of whom self-reported digestive symptoms with milk consumption [42] and 10 who were recruited as dairy tolerant controls, reporting no symptoms with milk consumption. The exclusion criteria included individuals with a BMI below 18 or above 28 kg/m^2^, use of antibiotics in the preceding 3 months, gastrointestinal disease (coeliac or inflammatory bowel diseases), or milk allergy. All participants provided written informed consent before the enrolment for the study and were compensated for their time. This study was conducted according to the Declaration of Helsinki and approved by the New Zealand Health and Disability Ethics Committees (Reference no.16/STH/175). Prospective clinical trial registration was registered at www.anzctr.org.au (ACTRN12616001694404). This study adheres to the CONSORT guidelines [43].

### Lactose and milk challenges

Prior to their visits, participants avoided dairy products for at least one week and fibre rich foods for 24 h. Following a standardized low fibre dinner and overnight fast of at least 8 h, participants attended the clinical unit in the morning on 3 occasions separated by at least 1 week. The study was conducted at the Liggins Institute, University of Auckland, between January and May 2017. A standardized lactose challenge was administered on the 1st visit which involved consumption of 50 g lactose (100% pure, Midwest Pharmaceutics, New Zealand) dissolved in 250 mL water. Although a lactose dose of 25 g is typical for a challenge [23–25] a higher load of 50 g [12, 22] confirms low lactase levels in lactose malabsorbers, without negatively affecting lactose absorbers [3]. On the subsequent three visits, subjects consumed, in a randomised sequence, 750 mL of a2 Milk™ (A2M) or conventional (CON) milk (both of which contained 37.5 g lactose) or lactose-free conventional milk (which contained both A1 and A2 β-casein but no lactose). However, this study reports secondary outcomes relevant only to lactose malabsorption and lactose containing milks, so does not report on data collected after the lactose-free milk. The sequence of milk treatment arms was randomly generated by www.randomizer.org, blocked by tolerance group, and sealed envelopes were used to allocate the treatment before the first milk tolerance test. Both the participants and researchers were blinded. For all four visits, blood, breath, and urine samples were collected from subjects prior to lactose or milk consumption (baseline) and at frequent intervals for 3 h. Exhaled breath was collected every 15 min until 2 h then every 30 min for 3 h; blood was collected every 30 min until 3 h, and urine was collected continuously for 3 h**.**

#### Blood collection and sampling

Venous blood was collected in EDTA containing tubes (Becton Dickinson & Company, Mount Wellington, New Zealand). Peripheral blood mononuclear cells (PBMCs) were extracted immediately from EDTA treated whole blood using Ficoll, as described previously [44] then stored at − 80 °C until DNA was extracted. Plasma was prepared from EDTA-treated whole blood through centrifugation at 2000×g for 15 min at 4 °C and frozen at − 80 °C prior to analyses.

#### Genetic test: C/T_13910_ and G/A_22018_ genotyping

DNA was isolated from PBMCs with the universal all Prep kit (Qiagen, Hilden, Germany) as per manufacturer’s protocol. LNP genotyping was determined by restriction fragment length polymorphism (RFLP) of polymerase chain reaction (PCR) amplified DNA. For C/T_13910_ forward primer 5′-GGACATACTAGAATTCACTGCAA and reverse primer 5′-GGTTGAAGCGAAGATGGGACG [45] were used. For G/A_22018_, the forward primer 5′-TAGCTGGGACCACAAGCACC and reverse primer 5′-GAAGTCAGAATACCCCTACCC were used as described [41]. The amplification product for C/T_13910_ was digested with *BsmF*1 (New England Biolabs, Foster City, CA) while G/A_22018_ was digested with *Hha*1 (New England Biolabs, Foster City, CA) as per the manufacturer’s protocol. The digested products along with the PCR products were visualized using ethidium bromide in 5% MetaPhor agarose gel (Lonza, Basel, Switzerland) and visualised using ethidium bromide. The results were confirmed by Sanger sequencing. Further details regarding the genotyping procedure are described elsewhere [41].

#### Malabsorption marker methods

Hydrogen in the exhaled breath was measured in parts per million (ppm) using a breath analyser (Quintron, Milwaukee, WI, USA). Plasma glucose and urinary creatinine were measured using a Roche Cobas C 311 by enzymatic colorimetric assay (Roche, Manheim, Germany). Urinary galactose was measured using the Amplex Red Galactase Oxidase Assay Kit (Molecular Probes, Eugene, Oregon, USA) according to the manufacturer’s protocol.

### Statistical analysis

Values are presented as mean ± SEM or 95% CI, as indicated. For all malabsorption markers, the change from baseline was calculated providing a single value for each subject. Comparisons between subject groups were computed by linear mixed model using subject as a random factor to account for repeated sampling as required. Outliers were identified as greater than Q3 + 3IQR. Statistical analyses were computed using R software (version 3.5.2) [46]. Alpha was set at 0.05.

Sample size was calculated for the primary outcome as described elsewhere [41]. Based on 80% power, α = 0.05, to detect an AUC = 0.80 [47], we determined that 10 cases and 10 controls would be sufficient. A similar study of diagnostic accuracy of a new test for diagnosing hypolactasia had 30 participants, but only with AUC = 0.75 [12].

#### LNP classification methods

Two polymorphisms (C/T_13910_ and G/A_22018_) of the LCT genotype were used as the reference standard for detecting LNP; binary classification was used to differentiate the LNP genotype (CC_13910_ / GG_22018_) from LP genotypes (TT_13910_ / AA_22018_ homozygotes and CT_13910_ / GA_22018_ heterozygotes) [12, 48]. The baseline characteristics between the LCT genotypes (homozygous LNP, homozygous LP and heterozygous LP) were compared using one-way analysis of variance (ANOVA).

#### Determination of validity of malabsorption marker method

The validity of the malabsorption marker methods was evaluated by the sensitivity, specificity, positive predictive value (PPV), negative predictive value (NPV), false positive (FP) and false negative (FN) [49]. The sensitivity of a test refers to the ability of the test to correctly classify an individual as diseased (true positive) whereas the specificity of a test refers to the ability of the test to detect individuals as disease-free (true negative) [50]. PPV is the probability that a subject with a positive test truly has the disease and NPV is the probability that a subject with a negative test truly does not have the disease [51]. FP is a type I error that indicates a disease exists when it actually does not. FN is a type II error that indicates a disease does not exist when it actually does [49].

#### Diagnostic accuracy and predicted cut-offs

Using the LCT genotype as the reference standard [12, 52] the diagnostic accuracy of the malabsorption marker methods was evaluated by receiver operating characteristic (ROC) curve as assessed by area under the ROC curve (AUC) using the pROC package in R software. A larger AUC indicates a better diagnostic outcome, such that 0.70 < AUC < 0.80 was considered 'acceptable', 0.80 < AUC < 0.90 as 'excellent' and AUC > 0.90 as 'outstanding' [47]. The discriminating ability of the test is determined when the ROC curve differs significantly (*p* < 0.05) from AUC 0.5 (i.e. no discrimination between true positive (TP; LNP) and true negative (TN; LP) [52]) using the Mann-Whitney U test. Differences between ROC curve AUCs were compared across substrates using the ROC test by bootstrap method. Optimal cut-off values using milk as a substrate were calculated by the Youden index for malabsorption markers with an acceptable AUC > 0.70.

#### Validity of literature cut-offs

To compare with literature, an increase over baseline in breath H_2_ ≥ 20 ppm blood glucose ≤1.11 mmol/L [12, 26] and postprandial galactose/creatinine ratio at ≤0.10 mg/mg [28] were taken as the cut-off values to diagnose LM (Table 1), and were then compared against the genotype results. For the standardised lactose challenge, classification of LNP and LP using malabsorption markers (breath H_2_, plasma glucose, and urinary galactose/creatinine ratio) was determined based on diagnostic cut-offs previously reported in literature (Table 1). Following milk consumption, cut-offs for breath H_2_, plasma glucose [32] and urinary galactose/creatinine ratio [31] were additionally adjusted for the dose of lactose consumed; this approach has previously been used in literature when using milk as a substrate [32, 35]. Similarly, as breath H_2_ has often been assumed to increase linearly with the dose of lactose consumed [23], the literature cut-off after milk was also adjusted for lactose dose: 15 ppm from 20 ppm, based on 37.5 g lactose in 750 mL of milk. The validity of the breath H_2_ was assessed with and without the adjusted value.

**Table 1:** Diagnostic cut-off for lactose malabsorption based on literature

Measure	Cut-off for lactose malabsorption^**1**^
Breath H_2_	≥20 ppm
Plasma glucose	≤1.11 mmol/L
Urinary galactose/creatinine ratio	≤0.10 mg/mg

Cut-off values are based on validated methods for maximal increase in breath H_2_ [23–25] and plasma glucose [12, 26] over baseline and urinary galactose/creatinine ratio [28] at 180 min, following ingestion of 50 g of lactose

The agreement between the genotype classification and literature-based malabsorption marker classification was determined using Cohen’s kappa coefficient.

## Results

### Participant characteristics

The majority of the participants were self-described as Caucasian (*n* = 25), with a minority of South Asian (*n* = 4), Chinese (*n* = 8), Māori (*n* = 1) and South African (n = 2) descent. There were no significant differences in age, BMI, glucose, insulin, and triglycerides at fasting between the three genotypes (*p* > 0.05, Table 2).

**Table 2:** Participant demographics

Measure^**a**^	LP genotypes^**b**^		LNP genotypes	***p*** value^**c**^
	LP Homozygote (*n* = 16) TT_13910_ / AA_22018_	LP Heterozygote (*n* = 10) CT_13910_ / GA_22018_	LNP Homozygote (*n* = 14) CC_13910_ / GG_22018_	
BMI (kg/m^2^)	22.5 ± 0.5	22.3 ± 0.5	24.4 ± 1.0	0.098
Age (years)	26.8 ± 0.6	24.2 ± 0.96	26.2 ± 0.68	0.060
Glucose (mmol/L)	5.52 ± 0.20	5.15 ± 0.16	5.5 ± 0.21	0.758
Triglycerides (mmol/L)	1.12 ± 0.11	1.11 ± 0.09	1.09 ± 0.09	0.965
Insulin (μU/mL)	13.39 ± 3.44	9.94 ± 1.5	13.64 ± 3.01	0.687

^a^ Glucose, triglycerides and insulin measures in plasma

^b^ Values presented as mean ± SEM. Genotypes for lactase persistence classification taken from [12, 48]

^c^*p* value was computed using linear mixed model

*BMI* body mass index, *LNP* lactase non-persistence, *LP* lactase persistence

### Lactase genotyping

There was 100% agreement between the C/T_13910_ and G/A_22018_ genotypes. Of the 40 individuals, 14 (35%) were LNP homozygotes (CC_13910_ / GG_22018_), and 26 were LP, consisting of 16 (40%) LP homozygotes (TT_13910_ / AA_22018_) and 10 (25%) LP heterozygotes (CT_13910_ / GA_22018_).

### Malabsorption marker difference between LP and LNP individuals with lactose or milk consumption

**Fig. 1:**
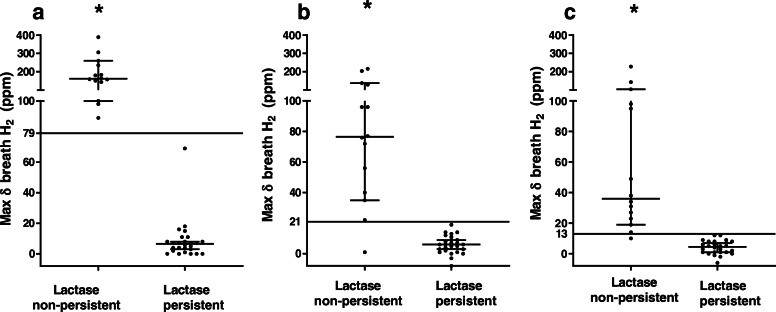
Increase in breath H_2_ in lactase persistent and lactase non–persistent individuals, who were identified by the genotyping test, after ingestion of (**a**) lactose (50 g), (**b**) conventional milk (CON; 750 mL; 37.5 g lactose) and (**c**) a2 Milk (A2M; 750 mL, 37.5 g lactose). * denotes *p *< 0.05 between LNP and LP. The cut-off value calculated from the ROC curve (Youden index) following ingestion of lactose, CON milk and A2M (79 ppm, 21 ppm and 13 ppm, respectively) is represented by a solid horizontal line for LP and LNP individuals for all three substrates (*p* < 0.001 each, respectively using Mann-Whitney U test). Group medians and 95% confidence intervals are denoted across data points

All the malabsorption markers measured (breath H_2_, plasma glucose and urinary galactose/creatinine ratio) differed between LP and LNP individuals (*p* < 0.05) after lactose. However, post milk challenge only breath H_2_ was different between LP and LNP individuals (*p* < 0.05), whereas plasma glucose and urinary galactose/creatinine ratio did not differ (*p* > 0.05) (Fig. 1 and Additional File 1: Table S1)). The kinetics of breath H_2,_ plasma glucose and urinary galactose/creatinine ratio between LP and LNP are shown in Additional File 2: Figure S1.

### Diagnostic accuracy of malabsorption marker using ROC curve analyses assessed against LCT genotypes

As the cut-off values described in literature for the malabsorption markers tested vary or have not been well established to specific lactose doses or substrates, the diagnostic accuracy of each malabsorption marker was assessed using ROC curve analyses (Table 3, Additional File 3: Figure S2).

**Table 3:** Accuracy of diagnostic measures and substrates for lactose malabsorption

Measure^**a**^ and substrate^**b**^	AUC^**c**^	95% CI	***p*** value^**d**^	Cut-off^**e**^	Sensitivity(%)	Specificity (%)
***Breath H***_***2***_						
Lactose	1.000	1	< 0.001	79 ppm	100	100
CON	0.942	0.828–1	< 0.001	21 ppm	92	100
A2M	0.994	0.981–1	< 0.001	13 ppm	92	100
***Plasma glucose***						
Lactose	0.755^a^	0.603–0.907	0.008	1.77 mmol/L	61	100
CON	0.535^b^	0.271–0.645	0.671	NA	NA	NA
A2M	0.458^b^	0.348–0.723	0.712	NA	NA	NA
***Urinary galactose/creatinine ratio***						
Lactose	0.758	0.611–0.905	0.008	0.03 mg/mg	53	100
CON	0.653	0.480–0.838	0.112	NA	NA	NA
A2M	0.618	0.433–0.803	0.223	NA	NA	NA

^a^Maximal increase in breath H_2_ and plasma glucose over baseline for 3 h, urinary galactose/creatinine ratio at 3 h post lactose and milk challenge

^b^ Lactose: 50 g lactose in 250 mL water; CON and A2M: 750 mL milk

^c^ Values are area under the receiver operating characteristics (ROC) curve (AUC)

Different letters (a, b) represent a significant difference in the AUCs of the ROC curve between substrates calculated by ROC test

^d^*p* value between calculated ROC AUC and AUC of 0.5 (no discrimination between true positives (TP; LNP) and true negatives (TN; LP)) was computed using the Mann-Whitney U test

^e^ Cut offs (calculated by the Youden Index) are presented, unless AUC > 0.7 and *P* value < 0.05

*A2M* a2 milk, *CON* conventional milk, *CI* confidence interval

#### Accuracy with 50 g lactose

Following lactose ingestion, breath H_2_ had ‘outstanding’ diagnostic accuracy with an AUC = 1.00. Comparatively, plasma glucose and urinary galactose/creatinine ratio accuracies were ‘acceptable’ (> 0.70) with AUC = 0.75 and 0.77 (Table 3). The AUC was significant for all three index measures after the lactose challenge (*p* < 0.01) (Table 3).

#### Accuracy with milk

Using milk as a substrate, the accuracy of breath H_2_ was ‘outstanding’ with AUC > 0.90 and *p* < 0.001 irrespective of the type of milk consumed (A2M or CON). However, glucose and galactose were ‘not reliable’ methods to diagnose LNP after milk ingestion with an AUC < 0.70 and *p* > 0.05 (Table 3), so the optimal cut-off for these measures could not be assessed.

#### ROC curve AUCs

The AUCs for breath H_2_ and urinary galactose/creatinine ratio did not differ between lactose or milk ingestion (*p* > 0.05). However, AUCs for glucose differed significantly between lactose and CON milk (*p* = 0.032) and between lactose and A2M (*p* = 0.042) (Table 3).

### Established cut-off value after lactose and milk challenge (A2M and CON Milk)

The optimal cut-off value for breath H_2_ after lactose and milk ingestion was determined by ROC curve analyses using the Youden index. The optimal cut-off for breath H_2_ after ingestion of lactose was 79 ppm providing sensitivity and specificity of 100%; when a statistical outlier was removed the cut off value was reduced to 53 ppm providing the same sensitivity and specificity. The cut-off value following CON milk ingestion was 21 ppm providing a sensitivity of 92% and specificity of 100% but for A2M it was 13 ppm, providing the same sensitivity and specificity. Following lactose ingestion, the cut-off values for plasma glucose and urinary galactose/creatinine ratio were 1.77 mmol/L and 0.03 mg/mg, respectively. Although the specificity remained high, sensitivity was lower for both plasma glucose and urinary galactose/creatinine than for breath H_2_ (Table 3). Since the AUCs for plasma glucose and urinary galactose/creatinine ratio following milk ingestion were < 0.7, the cut-off values were not calculated.

### Validity of malabsorption markers using literature cut-offs assessed against LCT genotype

#### Validity with lactose

Following lactose ingestion, the validity of malabsorption markers using literature cut-offs (Table 1) to diagnose LNP was assessed against the genotype reference standard. Breath H_2_ had 100% sensitivity and 96.2% specificity for LNP classification. Both the sensitivity and specificity for plasma glucose were below 70% (Table 4). The urinary galactose values were lower than previous reports, and no subject had a galactose/creatinine ratio above the literature cut-off of 0.1 mg/mg. Thus, urinary galactose/creatinine ratio failed to detect any LP individuals resulting in 100% sensitivity but 0% specificity for LNP.

**Table 4:** Performance of diagnostic measures of lactose malabsorption for detection of lactase non-persistence

Measure^a^ and substrate^b^	Cut-off used^c^	Sensitivity (%)	Specificity (%)	FP (n)	FN (n)	TP (n)	TN (n)	PPV (%)	NPV (%)	κ index^d^
***Breath H2***										
Lactose	20 ppm	100.0	96.2	1	0	14	25	93.3	100	0.9***
CON - unadjusted	20 ppm	92.9	100.0	0	1	13	26	100	96.3	0.9**
CON - adjusted	15 ppm	92.9	96.2	1	1	13	25	82.9	96.2	0.9***
A2M - unadjusted	20 ppm	78.6	100.0	0	3	11	26	100	89.6	0.8***
A2M - adjusted	15 ppm	85.7	100.0	0	2	12	26	100	89.7	0.8***
***Plasma glucose***										
Lactose	1.11 mmol/L	64.3	69.2	8	5	9	18	52.9	78.2	0.3*
CON - unadjusted	1.11 mmol/L	92.9	26.9	19	1	13	7	40.6	87.5	0.2
CON - adjusted	0.83 mmol/L	78.6	57.7	15	3	11	11	42.4	78.6	0.2
A2M - unadjusted	1.11 mmol/L	64.3	38.5	16	5	9	10	36	66.7	0.02
A2M - adjusted	0.83 mmol/L	57.1	46.2	14	6	8	12	36.4	77.8	0.02.
***Urinary galactose/creatinine ratio***^***e***^										
Lactose	0.10 mg/mg	100.0	NA	26	0	14	0	35	NA	0
CON- unadjusted	0.10 mg/mg	100.0	NA	26	0	14	0	35	NA	0
CON - adjusted	0.08 mg/mg	100.0	NA	26	0	14	0	35	NA	0
A2M- unadjusted	0.10 mg/mg	100.0	NA	26	0	14	0	35	NA	0
A2M- adjusted	0.08 mg/mg	100.0	NA	26	0	14	0	35	NA	0

Classification was based on the genotype reference standard for lactase non-persistence (LNP; n = 14) relative to lactase persistent (LP; *n* = 26)

^a^ Breath H_2_, plasma glucose indicates maximal increase in breath H_2_ and plasma glucose over baseline for 3 h post lactose and milk ingestion and urinary galactose/creatinine ratio at 3 h post lactose and milk ingestion

^b^ Lactose: 50 g lactose in 250 mL water; CON and A2M: 750 mL of milk

^c^Cut-off values for lactose and unadjusted (CON and A2M) were based on ingestion of 50 g lactose. Adjusted cut-off values were calculated based on ingestion of 750 mL of milk ~ 37.5 g of lactose

^d^κ represents the Cohen’s kappa index indicating the agreement between lactose malabsorption and genotype

Significance indicated as * *p* value < 0.05, ** *p* value < 0.01, *** *p* value < 0.001

^e^ Galactose/creatinine ratio concentration range was lower than the threshold of 0.10 mg/mg; no lactose malabsorption was detected by the method using the literature threshold of 0.10 mg/mg [28] for both CON and A2M

*A2M* a2 milk, *CON* conventional milk, *FN* false negative, *FP* false positive, *NPV* negative predictive value, *PPV* positive predictive value, *TN* true negative, *TP* true positive. FN and FP represent the total number of false negativeand false positive, respectively out of total (*N* = 40) participants

The agreement between the genetic classification and literature-based classification using breath H_2_ and plasma glucose was significant (*p* < 0.01; κ = 0.090 and κ = 0.319, *p* < 0.05 respectively), despite the lower sensitivity and specificity of the plasma glucose. Urinary galactose/creatinine ratio did not agree with the genetic reference standard due to the sample range falling below the literature cut-off.

#### Validity with milk

Following milk ingestion, the validity of malabsorption markers to correctly classify LNP was diminished across all malabsorption detection methods used. Validity was assessed using the literature reported cut-offs with and without adjusting for the dose of lactose (Table 4).

The sensitivity of breath H_2_ was unchanged (i.e. 92.9%) with or without the adjusted H_2_ value following CON milk ingestion whereas sensitivity was improved following A2M ingestion with the adjusted H_2_ value from 78.6 to 85.7%. However, following CON milk ingestion, the specificity decreased from 100 to 96.2% with the adjusted H_2_ value although specificity remained unchanged for A2M (100%). The specificity for plasma glucose was < 58% with more than 13 false positive results for both types of milk with or without adjustment for lactose dose. However, the specificity was improved from 26.9 to 57.7% (CON milk) and 38.5 to 46.2% (A2M) after the adjustment. The sensitivity was slightly reduced from (64.3% to 57.1) following A2M ingestion but moreso for CON milk (from 92.9 to 78.6%) after the adjustment. For the urinary galactose/creatinine ratio, the concentration was lower than the optimal cut-off with or without adjustments.

## Discussion

This study demonstrated that following either a lactose or milk challenge breath H_2_ was a reliable method to detect LNP, showing strong agreement (100%) with genetic polymorphic analysis. Breath H_2_ was superior to either plasma glucose or urinary galactose/creatinine ratio in the assessment of LNP after both lactose and milk challenge. A marked reduction in accuracy of plasma glucose and urinary galactose/creatinine was observed when milk was used as the challenge substrate. The cut-off calculated after lactose challenge for both breath H_2_ and plasma glucose was higher (79 ppm and 1.77 mmol/L) than the literature cut-offs (20 ppm [23–25] and 1.11 mmol/L [12, 26]) whereas urine galactose concentrations were generally 10 fold lower than concentrations previously reported, probably due to variation in the method used resulting in a lower threshold (0.03 mg/mg) than previously reported (0.10 mg/mg) [28]. Furthermore, this study demonstrated that β-casein variation in milk was a further determinant of the breath H_2_ test performance. A2M exhibited a lower optimal breath H_2_ threshold for accurate LNP discrimination than CON milk which corresponds to a lower rise in breath H_2_ observed after A2M compared to CON milk [41].

Breath H_2_ after a lactose challenge provided a very high sensitivity and specificity to diagnose LNP. This is consistent with previously reported high agreement (90%) between breath H_2_ and C/T_13910_ genotype, with 97% specificity and 95% sensitivity (where breath H_2_ was the reference standard) [50]. In the current study, plasma glucose exhibited low sensitivity and specificity, consistent with previous reports [11]. Glucose regulation in the body is complex and influenced by gastric emptying, hormonal regulation [10], and absorbed carbohydrate, which may explain the method’s lower accuracy. Rapid insulin responses to meals have been shown to result in false positive results, whereas in diabetic patients impaired glucose homeostasis may give false negative results [53]. Similar to glucose, the urinary galactose/creatinine ratio was less accurate than breath H_2_ as urinary galactose may also be affected by gastrointestinal, hepatic and renal metabolism [29], or by the urine adjustment method (no adjustment [31], creatinine [28], or specific gravity [54]). Moreover, the sensitivity and specificity of urinary galactose could not be reliably assessed due to differences in detection ranges from previous reports [27–29], highlighting the variability of this method. Thus, plasma glucose and urinary galactose are less preferred methods to diagnose LNP with lactose than breath H_2_.

The accuracy of plasma glucose and urinary galactose/creatinine ratio in diagnosing LNP after a milk challenge has not yet been reported, although both are used in practice [32, 35]. Based on our ROC curve analysis following milk ingestion, the AUCs for plasma glucose and urinary galactose/creatinine ratio were unreliable (AUC < 0.70) and could not differentiate LP from LNP. The presence of fats and protein in milk is likely to interfere with glucose and galactose metabolism, impacting these measures. The postprandial glucose response to carbohydrate ingested with fats may reduce glucose responses due to fat-induced delayed gastric emptying [55]. Indeed, the blood glucose response to lactose was previously shown to be lower with whole and skim milk than isolated lactose [56]. Studies in rodents showed lowered galactosuria when fat was included in lactose-containing diets [57, 58]. These digestive and metabolic influences may explain the poorer accuracy of plasma glucose and urinary galactose/creatinine to reliably diagnose LNP after milk ingestion; the greater risk of false positive results with these methods should be considered prior to their use.

It is important to note that the change in breath H_2_ depends on the dose of the lactose administered [23]. Lactose doses vary within clinical and research practice, ranging from adjustments per kg body weight (particularly for children) [59], to standard doses used to ensure LM (50 g) [22, 60], or to approximate realistic doses (e.g., 10 g [61] to 25 g [62]). However, the cut-off value is not always dose-adjusted to diagnose LM or LNP. Where milk is used as a substrate, the dose of lactose is likely to be lower, as one serving (250 mL) of milk has only 12 g lactose. Our study demonstrated that without a lactose-dose adjustment of the cut-off, the sensitivity of breath H_2_ using A2M was reduced to 78.6%, more similar to the sensitivities observed for plasma glucose. With a lactose-dose adjustment of the cut-off, the sensitivity after A2M was improved due to avoidance of one false negative result. However, lactose-dose adjustment did not improve the breath H_2_ sensitivity following CON ingestion, and rather decreased specificity to 96.2% due to one false positive. This difference between milks was also evident with the lactose-dose adjustment cut-off for A2M (15 ppm) more closely approximating the calculated cut-off (13 ppm), while for CON some discrepancy remained. Hence, to preserve the sensitivity and specificity of LM detection, even using the highly reliable breath H_2_, cut-off values should be used that have been validated for the specific dose and substrate of lactose used.

The breath H_2_ cut-off values calculated from this study, especially after a lactose challenge, were much higher than the literature cut-offs. One reason could be that one LP heterozygous individual had much higher breath H_2_ than the rest of the group (i.e. outlier); yet, even when this was accounted for, the breath H_2_ cut-off still remained higher than the literature cut-offs. The calculated cut-off may also have been high due to the higher lactose dose used, or the presence of high-grade lactose malabsorbers (breath H_2_ increase > 70 ppm) [63]. The use of literature cut-offs however detected lactose malabsorption with 100% sensitivity, with presence of only 1 FP (which was the heterozygous LP individual). Thus in order to achieve higher sensitivity it is appropriate to use a lower cut off (20 ppm) after lactose challenge.

The milk types used in this study contained either A1 and A2 β-casein (CON milk) or exclusively A2 β-casein (A2M). Despite having the same quantity of lactose, these milk types resulted in different breath H_2_ concentrations, different sensitivity for breath H_2_ to detect LNP and different calculated optimal cut-off values (CON: 21 ppm vs A2M: 13 ppm). Although milk has been used as a substrate for lactose tolerance testing [32, 35], differences compared to isolated lactose on malabsorption markers [22, 56] and the impact of variability of components within milk [56] is not widely reported. The lower sensitivity and cut-off for A2M suggests that the A1 β-casein content of milk may influence the H_2_ concentration in breath, which may be a factor to consider when milk is used as a substrate to diagnose LNP using the H_2_ breath test. The mechanisms leading to a higher cut-off for malabsorption with A1 β-casein were not determined in the current study, but could be due to gastrointestinal transit differences between A1 and A2 β-casein [31, 64], or peptide-mediated mechanisms [65]. The A1 β-casein content of milk differs between breeds and herds of cows, such that milk produced by pure Asian or African breeds [40] (including Jersey and Guernsey [66]) are usually free from A1 β-casein (like A2M), unlike European breeds [40] including Holstein Friesian [66] (which are more like CON milk with both A1 and A2 β-casein). Although A2 gene frequency is higher in Jersey cows (more than 50%) [67] this may vary between herds [66]. Depending on the region, breed, or even the herd that milk is sourced from, these types of protein variability may impact the cut-off required for accurate diagnosis. This variability suggests that cut-offs specific to the milk substrate used are required, in lieu of a universal optimal value for LM or LNP. Nevertheless, the added risk of false negatives or false positives using alternate substrates should be acknowledged prior to testing.

Two polymorphisms upstream of the *LCT* gene (C/T_13910_ and G/A_22018_) were used as the reference standard to diagnose LNP [14, 15]. This method does not detect secondary LM originating from intestinal damage and may be inaccurate for certain populations [16]. The C/T_13910_ polymorphism has been studied predominantly in Caucasian populations [13], while the G/A_22018_ polymorphism is more prominent in Northern Chinese [68] and Indian populations [15]. As there is limited information on LP SNPs among non-Caucasian populations, [68] the selection of SNPs, along with the limited diversity, small sample size, and female-only subject pool, remains a limitation for the generalisability of this study. Further, although lactase activity remains with a single LP allele [69] supporting the binary classification of CT/GA heterozygotes as LP in the current study, it has been reported that CT heterozygotes have an intermediate level of lactase activity, higher than CC homozygotes and lower than TT homozygotes [14, 70, 71]. This effect has been shown to result in higher breath H_2_ in CT_13910_ heterozygotes compared to TT_13910_ homozygotes [70] and could explain the lower correlation between LP (C/T_13910_ and TT_13910_) and breath H_2_ compared to the CC_13910_ genotype reported by others [72]. Thus, genotyping combined with a breath H_2_ test is likely to provide the best diagnosis of LNP as an alternative to lactase activity in the intestine.

## Conclusion

Breath hydrogen was more reliable to diagnose LNP compared to plasma glucose and urinary galactose/creatinine ratio after a lactose challenge. When milk was used as a substrate, breath H_2_ was a reliable method to diagnose LNP but plasma glucose and urinary galactose/creatinine ratio were not reliable when using available cut-off values. However, breath H_2_ accuracy and optimal cut-off values may depend on the protein content in the milk, as higher A2 β-casein content reduced the diagnostic threshold relative to lower A2 β-casein content or lactose alone. Therefore, the diagnostic threshold of even highly reliable malabsorption tests like breath H_2_ may vary depending on the substrate used.

## Additional Files

**Additional File 1: Table S1.** Response to lactose and milk ingestion. Table showing group means (lactase persistent and lactase non-persistent) means of breath H_2_ concentration, plasma glucose concentration, and urinary galactose/creatinine ratio following lactose (50 g), conventional milk (750 mL), or a2 Milk™ (750 mL).

**Additional File 2: Figure S1.** Pre and postprandial concentration of A) breath H_2_, B) plasma glucose and C) urinary galactose/creatinine between lactase persistent and lactase non-persistent individuals following ingestion of lactose, CON milk, and A2M. A) and B) show the timecoure change in breath H2 and plasma glucose respectively pre and post lactose and milk ingestion. C) shows the urinary galactose/creatinine concentration pre and post lactose and milk ingestion. Comparisons computed by generalised linear mixed model. Interaction between group and time are shown on each plot. * *p* < 0.05 between groups as denoted at each timepoint, or across a range of timepoints as indicated.

**Additional File 3: Figure S2.** The receiver operating characteristic (ROC) curve for breath H_2_, plasma glucose, and urinary galactose/creatinine ratio.

## Data Availability

Data described in the manuscript, code book, and analytic code will not be made available because approval has not been granted by subjects.

## Abbreviations

AUC: Area under the curve
FN: False negative
FP: False positive
LM: Lactose malabsorption
LNP: Lactase non-persistence
LP: Lactase persistence
LI: Lactose intolerance
NPV: Negative predictive value
PBMC: Peripheral blood mononuclear cells
PCR: Polymerase chain reaction
PPV: Positive predictive value
ROC: Receiver operating characteristics
SNP: Single nucleotide polymorphism
