# Supplementary material for: Evaluation of breath, plasma, and urinary markers of lactose malabsorption to diagnose lactase non-persistence following lactose or milk ingestion
Source: BMC Gastroenterol. 2020 Jun 29;20:204. doi: 10.1186/s12876-020-01352-6 (PMC7325051; doi:10.1186/s12876-020-01352-6)
Supplement: Supplementary file 1 — Additional File 1: Table S1. Response to lactose and milk ingestion. Table showing group means (lactase persistent and lactase non-persistent) means of breath H2 concentration, plasma glucose concentration, and urinary galactose/creatinine ratio following lactose (50 g), conventional milk (750 mL), or a2 Milk™ (750 mL). [file 12876_2020_1352_MOESM1_ESM.docx]

**Additional file 1**

| **Measures^1^ and substrate^2^** | **Lactase Non-Persistent (LNP)^3^**  **n = 14** | **Lactase Persistent (LP)^3^ n = 26** | ***P* value^4^** |
| --- | --- | --- | --- |
| ***Breath H_2_ (ppm)*** |  |  |  |
| Lactose | 186.86 ± 22.62 | 8.5 ± 2.62 | <0.001 |
| CON | 89.86 ± 16.62 | 5.93 ± 0.86 | <0.001 |
| A2M | 65.22 ± 17.02 | 4.12 ± 1.16 | <0.001 |
|  |  |  |  |
| ***Plasma glucose (mmol/L)*** |  |  |  |
| Lactose | 0.87 ± 0.12 | 1.93 ± 0.24 | 0.008 |
| CON | -0.52 ± 0.26 | -0.75 ± 0.22 | 0.671 |
| A2M | -0.62 ± 0.19 | -0.99 ± 0.2 | 0.712 |
| ***Urinary galactose /creatinine (mg/mg)*** |  |  |  |
| Lactose | 0.01 ± 0.01 | 0.04 ± 0.01 | 0.008 |
| CON Milk | 0.01 ± 0.01 | 0.02 ± 0.01 | 0.112 |
| A2M | 0.02 ± 0.01 | 0.02 ± 0.01 | 0.223 |

**Table S1.** **Response to lactose and milk ingestion**

^1^Maximal increase in breath H_2_ and plasma glucose over baseline for 3 h, urinary galactose/creatinine ratio at 3 h post lactose and milk challenge

^2^ Lactose: 50g lactose; CON and A2M: 750 mL milk.

^3^ Values are mean and standard error of mean (SEM).

^4^ *P* value was computed using Mann-Whitney U test.
