# Supplementary material for: Evaluation of breath, plasma, and urinary markers of lactose malabsorption to diagnose lactase non-persistence following lactose or milk ingestion
Source: BMC Gastroenterol. 2020 Jun 29;20:204. doi: 10.1186/s12876-020-01352-6 (PMC7325051; doi:10.1186/s12876-020-01352-6)
Supplement: Supplementary file 2 — Additional File 2: Figure S1. Pre and postprandial concentration of A) breath H2, B) plasma glucose and C) urinary galactose/creatinine between lactase persistent and lactase non-persistent individuals following ingestion of lactose, CON milk, and A2M. A) and B) show the timecoure change in breath H2 and plasma glucose respectively pre and post lactose and milk ingestion. C) shows the urinary galactose/creatinine concentration pre and post lactose and milk ingestion. Comparisons computed by generalised linear mixed model. Interaction between group and time are shown on each plot. * p < 0.05 between groups as denoted at each timepoint, or across a range of timepoints as indicated. [file 12876_2020_1352_MOESM2_ESM.pdf]

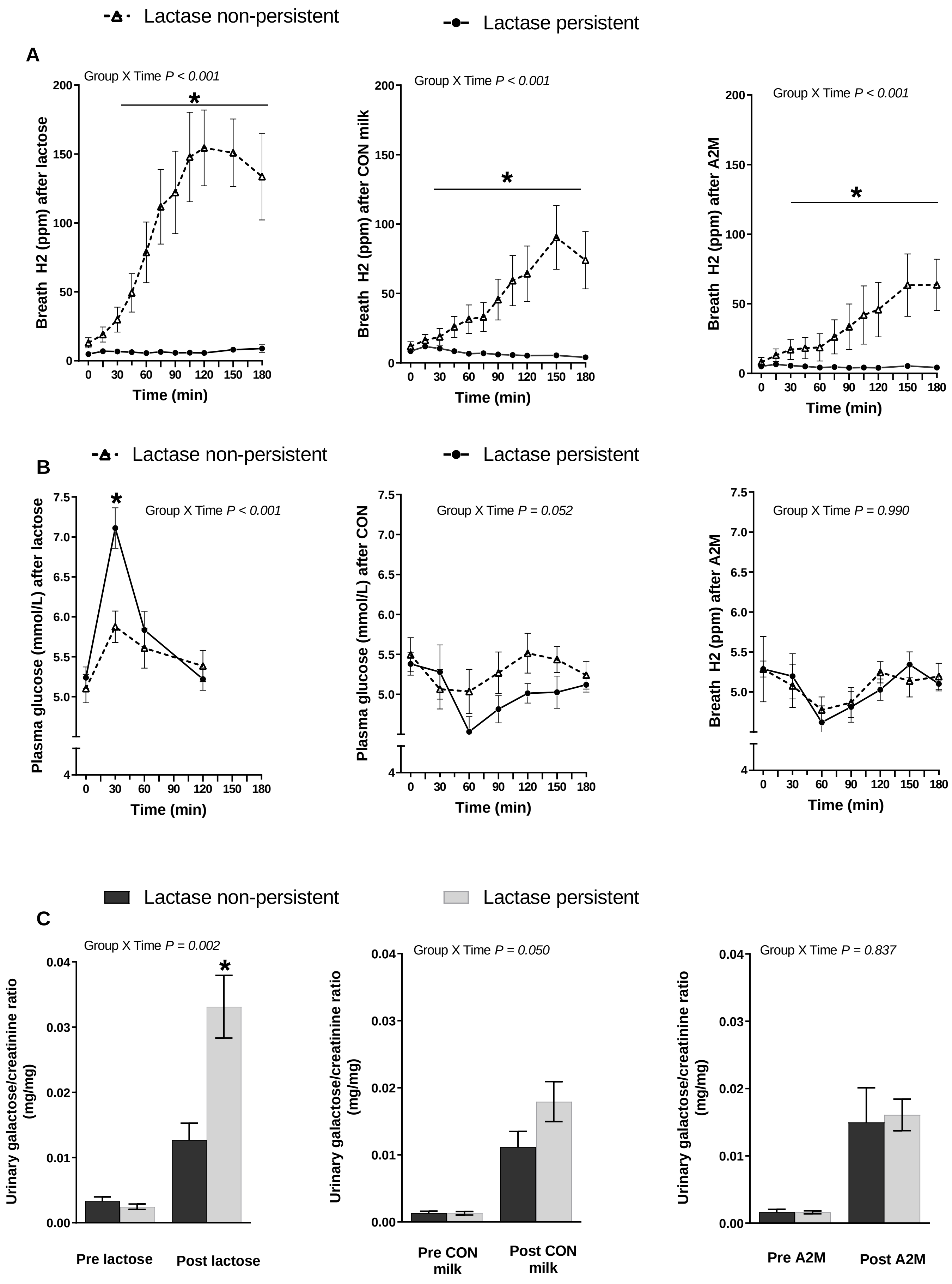
Lactase non-persistent
Lactase persistent
A
Group X Time P < 0.001
Breath H2 (ppm) after lactose
Time (min)
Group X Time P < 0.001
Breath H2 (ppm) after CON milk
Time (min)
Group X Time P < 0.001
Breath H2 (ppm) after A2M
Time (min)
Lactase non-persistent
Lactase persistent
B
Group X Time P < 0.001
Plasma glucose (mmol/L) after lactose
Time (min)
Group X Time P = 0.052
Plasma glucose (mmol/L) after CON
Time (min)
Group X Time P = 0.990
Breath H2 (ppm) after A2M
Time (min)
Lactase non-persistent
Lactase persistent
C
Group X Time P = 0.002
Urinary galactose/creatinine ratio (mg/mg)
Pre lactose
Post lactose
Group X Time P = 0.050
Urinary galactose/creatinine ratio (mg/mg)
Pre CON milk
Post CON milk
Group X Time P = 0.837
Urinary galactose/creatinine ratio (mg/mg)
Pre A2M
Post A2M
