# Supplementary figures and images for: Evaluation of breath, plasma, and urinary markers of lactose malabsorption to diagnose lactase non-persistence following lactose or milk ingestion
Source: BMC Gastroenterol. 2020 Jun 29;20:204. doi: 10.1186/s12876-020-01352-6 (PMC7325051; doi:10.1186/s12876-020-01352-6)

— Lactose  
- - - Conventional milk  
..... A2 milk

Breath H2

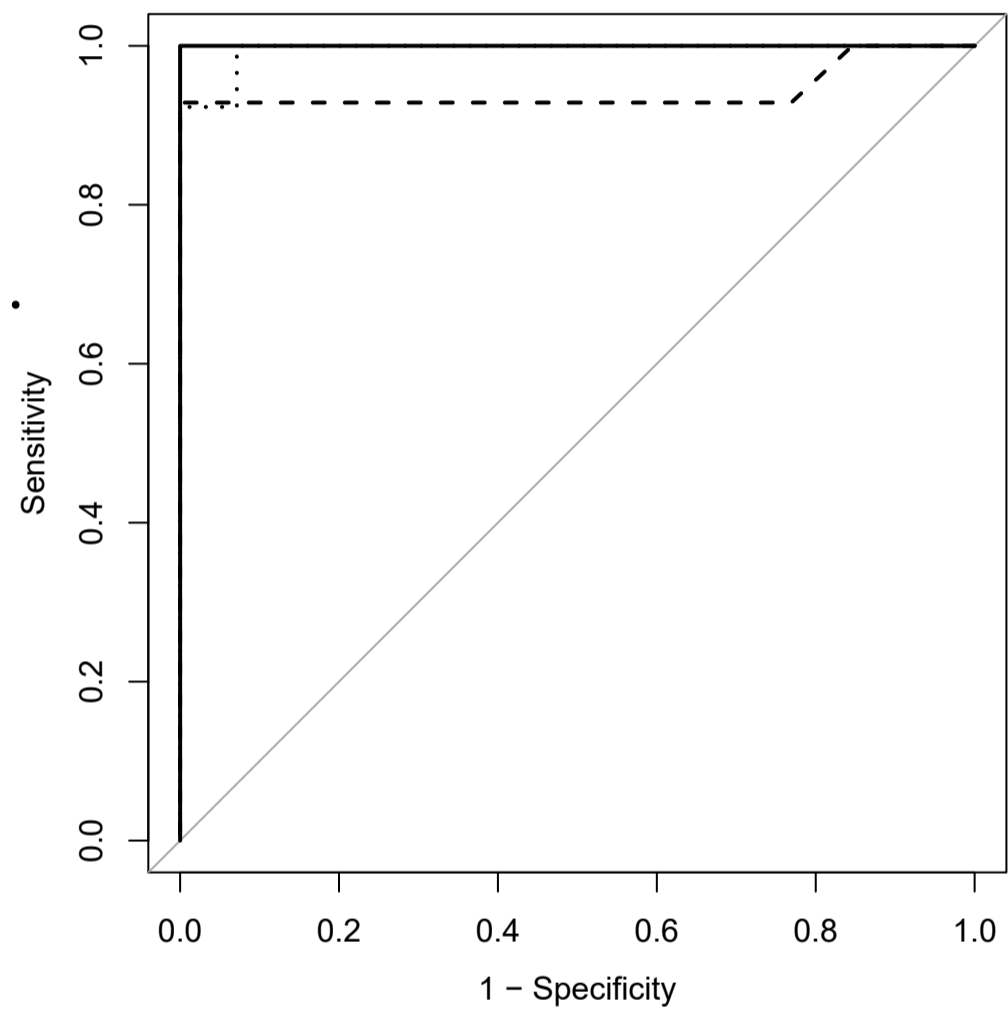

Plasma glucose

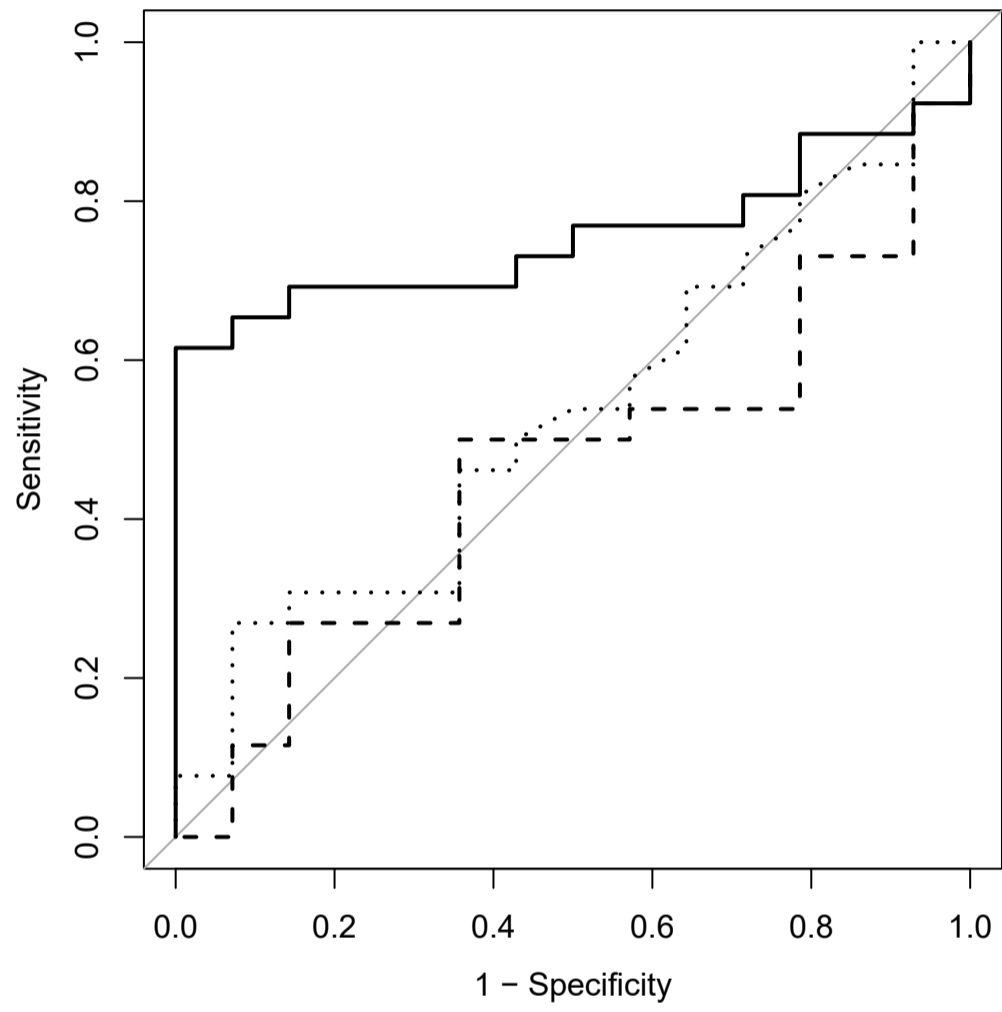

Urinary galctose/creatinine

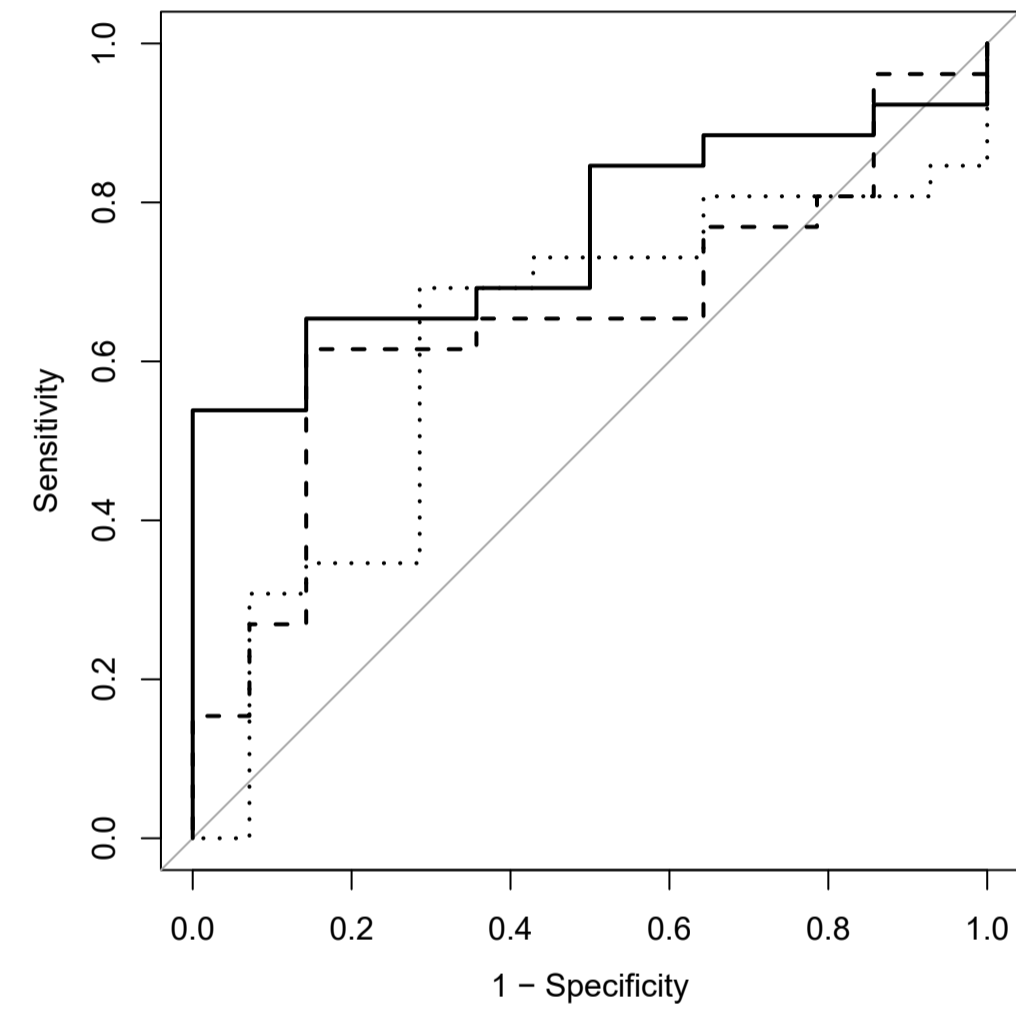

Supplement: Supplementary file 3 — Additional File 3: Figure S2. The receiver operating characteristic (ROC) curve for breath H2, plasma glucose, and urinary galactose/creatinine ratio. [file 12876_2020_1352_MOESM3_ESM.pdf]
